# Supplementary material for: Implementation of Medical Hospitalist Care at a Korean Tertiary Hospital: A Retrospective Cross-Sectional Study
Source: J Clin Med. 2024 Oct 28;13(21):6460. doi: 10.3390/jcm13216460 (PMC11547060; doi:10.3390/jcm13216460)
Supplement: Supplementary file 1 [file jcm-13-06460-s001.zip › jcm-3276541-supplementary.pdf]

SUPPLEMENTARY INFORMATION

TITLE: Implementation of Medical Hospitalist Care at a Korean Tertiary Hospital: A Retrospective Cross-Sectional Study

Authors:

Han Sung Lee, Seung Kyo Park, Sung Woo Moon

Supplementary Table S1. Final Diagnosis as ICD-10 Code for Each Major Cause of Admission

Major Cause of admission	Final diagnosis as ICD-10 Code	Number of patients
1. GI	Certain infectious and parasitic diseases (A00-B99)	85
	Neoplasms (C00-D48)	107
	Diseases of the digestive system (K00-K93)	631
	Etc.	29
2. pulmonary	Certain infectious and parasitic diseases (A00-B99)	75
	Neoplasms (C00-D48)	44
	Diseases of the circulatory system (I00-I99)	26
	Diseases of the respiratory system (J00-J99)	1,121
	Etc.	20
3. Cardiology	Diseases of the blood and blood-forming organs and certain disorders involving the immune mechanism (D50-D89)	8
	Diseases of the circulatory system (I00-I99)	135
	Diseases of the respiratory system (J00-J99)	27
	Injury, poisoning and certain other consequences of external causes (S00-T98)	20

	Etc	18
4. Hemato-oncology	Neoplasms<br>(C00-D48)	76
	Diseases of the blood and blood-forming organs and certain disorders involving the immune mechanism<br>(D50-D89)	71
	Symptoms, signs and abnormal clinical and laboratory findings, not elsewhere classified<br>(R00-R99)	6
	Etc.	6
5. Nephrology	Endocrine, nutritional and metabolic diseases<br>(E00-E90)	146
	Diseases of the musculoskeletal system and connective tissue<br>(M00-M99)	23
	Diseases of the genitourinary system<br>(N00-N99)	589
	Etc.	28
6. Infectious diseases	Certain infectious and parasitic diseases<br>(A00-B99)	138
	Diseases of the skin and subcutaneous tissue<br>(L00-L99)	34
	Diseases of the musculoskeletal system and connective tissue<br>(M00-M99)	32
	Symptoms, signs and abnormal clinical and laboratory findings, not elsewhere classified<br>(R00-R99)	111
	Codes for special purposes<br>(U00-U85)	22
	Etc.	31
7. Other diseases	Endocrine, nutritional and metabolic diseases<br>(E00-E90)	179
	Diseases of the nervous system<br>(G00-G99)	23
	Diseases of the circulatory system<br>(I00-I99)	31
	Diseases of the skin and subcutaneous tissue<br>(L00-L99)	12

	Diseases of the musculoskeletal system and connective tissue<br>(M00-M99)	20
	Symptoms, signs and abnormal clinical and laboratory findings, not elsewhere classified<br>(R00-R99)	21
	Injury, poisoning and certain other consequences of external causes<br>(S00-T98)	42
	Etc.	16
